# Genetic etiologies of the electrical status epilepticus during slow wave sleep: systematic review

**DOI:** 10.1186/s12863-018-0628-5

**Published:** 2018-07-06

**Authors:** Miriam Kessi, Jing Peng, Lifen Yang, Juan Xiong, Haolin Duan, Nan Pang, Fei Yin

**Affiliations:** 1 Department of Pediatrics, Xiangya Hospital, Central South University, 87 Xiang Ya Road, Changsha, 410008 Hunan Province China; 2 Kilimanjaro Christian Medical University College, 2240 Moshi, Tanzania

**Keywords:** Electrical status epilepticus during slow-wave sleep, Continuous spike-wave of slow sleep, Epilepsy aphasia spectrum, Monogenic mutations, Copy number variations, Channelopathy, Review

## Abstract

**Background:**

Electrical status epilepticus during slow-wave sleep (ESESS) which is also known as continuous spike-wave of slow sleep (CSWSS) is type of electroencephalographic (EEG) pattern which is seen in ESESS/CSWSS/epilepsy aphasia spectrum. This EEG pattern can occur alone or with other syndromes. Its etiology is not clear, however, brain malformations, immune disorders, and genetic etiologies are suspected to contribute. We aimed to perform a systematic review of all genetic etiologies which have been reported to associate with ESESS/CSWSS/epilepsy-aphasia spectrum. We further aimed to identify the common underlying pathway which can explain it. To our knowledge, there is no available systematic review of genetic etiologies of ESESS/CSWSS/epilepsy-aphasia spectrum. MEDLINE, EMBASE, PubMed and Cochrane review database were searched, using terms specific to electrical status epilepticus during sleep or continuous spike–wave discharges during slow sleep or epilepsy-aphasia spectrum and of studies of genetic etiologies. These included monogenic mutations and copy number variations (CNVs). For each suspected dosage-sensitive gene, further studies were performed through OMIM and PubMed database.

**Results:**

Twenty-six studies out of the 136 identified studies satisfied our inclusion criteria. I51 cases were identified among those 26 studies. 16 studies reported 11 monogenic mutations: *SCN2A* (N = 6), *NHE6*/*SLC9A6* (N = 1), *DRPLA*/ *ATN1* (N = 1), Neuroserpin/*SRPX2* (N = 1), *OPA3* (N = 1), *KCNQ2* (N = 2)*, KCNA2* (N = 5), *GRIN2A* (N = 34), *CNKSR2* (N = 2), *SLC6A1* (N = 2) and *KCNB1* (N = 5). 10 studies reported 89 CNVs including 9 recurrent ones: Xp22.12 deletion encompassing *CNKSR2* (N = 6), 16p13 deletion encompassing *GRIN2A* (N = 4), 15q11.2–13.1 duplication (N = 15), 3q29 duplication (N = 11), 11p13 duplication (N = 2), 10q21.3 deletion (N = 2), 3q25 deletion (N = 2), 8p23.3 deletion (N = 2) and 9p24.2 (N = 2). 68 of the reported genetic etiologies including monogenic mutations and CNVs were detected in patients with ESESS/CSWSS/epilepsy aphasia spectrum solely. The most common underlying pathway was channelopathy (N = 56).

**Conclusions:**

Our review suggests that genetic etiologies have a role to play in the occurrence of ESESS/CSWSS/epilepsy-aphasia spectrum. The common underlying pathway is channelopathy. Therefore we propose more genetic studies to be done for more discoveries which can pave a way for proper drug identification. We also suggest development of common cut-off value for spike-wave index to ensure common language among clinicians and researchers.

**Electronic supplementary material:**

The online version of this article (10.1186/s12863-018-0628-5) contains supplementary material, which is available to authorized users.

## Background

Electrical status epilepticus during slow-wave sleep (ESESS) which is also known as continuous spike-wave of slow sleep (CSWSS) is a type of an electroencephalogram (EEG) pattern which is characterized by presence of generalized bilateral and symmetric 1.5–3 Hz spike-waves [1]. The International League against Epilepsy (ILAE) defined it as the presence of significant activation of epileptiform discharges during sleep but no specific cut off-value for spike-wave index was indicated (Commission on Classification and Terminology of the International League Against Epilepsy 1989). Some authors have suggested the cut-off value of at least 85% [2, 3] but others have set the cut-off value at different percentage levels. This brings contradiction among clinicians, for instance Fernández IS et al. found in their survey that, 57.6% of the members of the Child Neurology Society and the American Epilepsy Society defined it by considering a cut-off value of 85% spike-wave index while 30.8% considered a cut-off value of 50% [4]. The ESESS/CSWSS pattern can be seen in different electroclinical syndromes with similar presentation including ESESS/CSWSS/epilepsy-aphasia spectrum. ESESS/CSWSS/epilepsy-aphasia spectrum is an acquired condition characterized by a triad of epilepsy, cognitive or behavioral impairment, and EEG abnormality of ESESS/CSWSS [1, 3, 5]. Epilepsy-aphasia spectrum is a spectrum of disorders ranging from severe epileptic encephalopathy with CSWSS and Landau- Kleffner syndrome (LKS) to the mild condition of childhood epilepsy with centrotemporal spikes [6–8]. ESESS/CSWSS/epilepsy-aphasia spectrum is age related and occurs commonly during the childhood usually in the first decade of life. It has a prevalence of about 0.5% of all childhood epilepsies [9]. It has long-term poor prognosis due to the persistence of neuropsychological impairment. Despite the fact that the ESESS/CSWSS pattern can be seen in ESESS/CSWSS/epilepsy-aphasia spectrum, it can also concur with other syndromes such as Rett syndrome, Costeff syndrome, Christianson syndrome, Tuberous sclerosis complex, Cohen syndrome and autism spectrum disorders [10–15].

The underlying etiology is unknown although brain malformations, immune disorders, and genetic factors have been reported. Brain malformations include porencephaly, polymicrogyria, pachygyria, cortical atrophy, and hydrocephalus [3, 9, 10, 16]. Immunity disorders with evidence of onconeuronal antibodies have been reported [17, 18]. Furthermore, few genetic causes have been reported including monogenic mutations and copy number variations (CNVs) [12, 19, 20]. Despite the availability of advanced technology in cytogenetic tests, few studies have focused on patients with ESESS/CSWSS/epilepsy-aphasia spectrum and the underlying mechanism for its occurrence remains unknown.

We aimed to perform a systematic review on all reported genetic etiologies of ESESS/CSWSS/epilepsy-aphasia spectrum including monogenic mutations and copy number variations. We further aimed to study the possible underlying mechanism for all reported genetic abnormalities especially for those associated with ESESS/CSWSS/epilepsy-aphasia spectrum solely. We believe this will help to identify the common genetic etiologies which can pave the way for the development of the appropriate therapy. This will help to reduce the burden of the complication of ESESS/CSWSS/epilepsy-aphasia spectrum owing to its long-term poor prognosis due to the persistence of neuropsychological impairment. Furthermore, our review will discover the existing gap and provide some suggestions. To our knowledge, there is no systematic review which has been done on the genetic etiologies of ESESS/CSWSS/epilepsy-aphasia spectrum.

## Methods

### Selection of studies

We developed search strategies for studies on genetic etiologies of electrical status epilepticus during sleep or continuous spike–wave discharges during slow sleep or epilepsy-aphasia spectrum in consultation with a librarian (Additional file 1). MEDLINE, EMBASE, PubMed and Cochrane review database were searched, using terms specific to electrical status epilepticus during sleep or continuous spike–wave discharges during slow sleep or epilepsy-aphasia spectrum and of studies of genetic etiologies. Studies reporting the monogenic mutations or copy number variations related to electrical status epilepticus during sleep or continuous spike–wave discharges during slow sleep were included. We included the studies which were done in human beings in all ages all over the world. We included case reports, case series, and cohort studies. Studies done in all years were included. Three independent reviewers screened the abstracts to determine if a full-text review should be performed. We included the studies published in English only and original peer-reviewed articles. We further performed hand searching of the references of articles that met eligibility criteria to ensure that additional relevant studies were not missed. We excluded the animal studies.

### Data extraction

Data extraction for all studies was performed by three independent reviewers, and the accuracy of information extracted was confirmed by discussion. Collected data related to monogenic mutations included; gene information such as name/alternate name, gene location, the Online Mendelian Inheritance in Man (OMIM) number, type of mutation, the function of the gene, the possible underlying pathway, the number of reported cases, associated syndromes, authors and the years for publication. Collected information related to copy number variations included; the chromosomal location, coordinates when available, type of aberration, size, gene content, gene of interest for ESESS/ CSWSS, the possible underlying pathway, the number of reported cases, associated syndromes, authors and the years for publication. All the suspected pathogenic genes were further studied individually in OMIM and PubMed database to determine their functions and how do they relate to ESESS/CSWSS. Additionally, we collected information related to the diagnostic criteria (spike wave index) which was used to diagnose ESESS/CSWSS/epilepsy-aphasia spectrum.

## Results

### Results of the systematic literature review

**Fig. 1:**
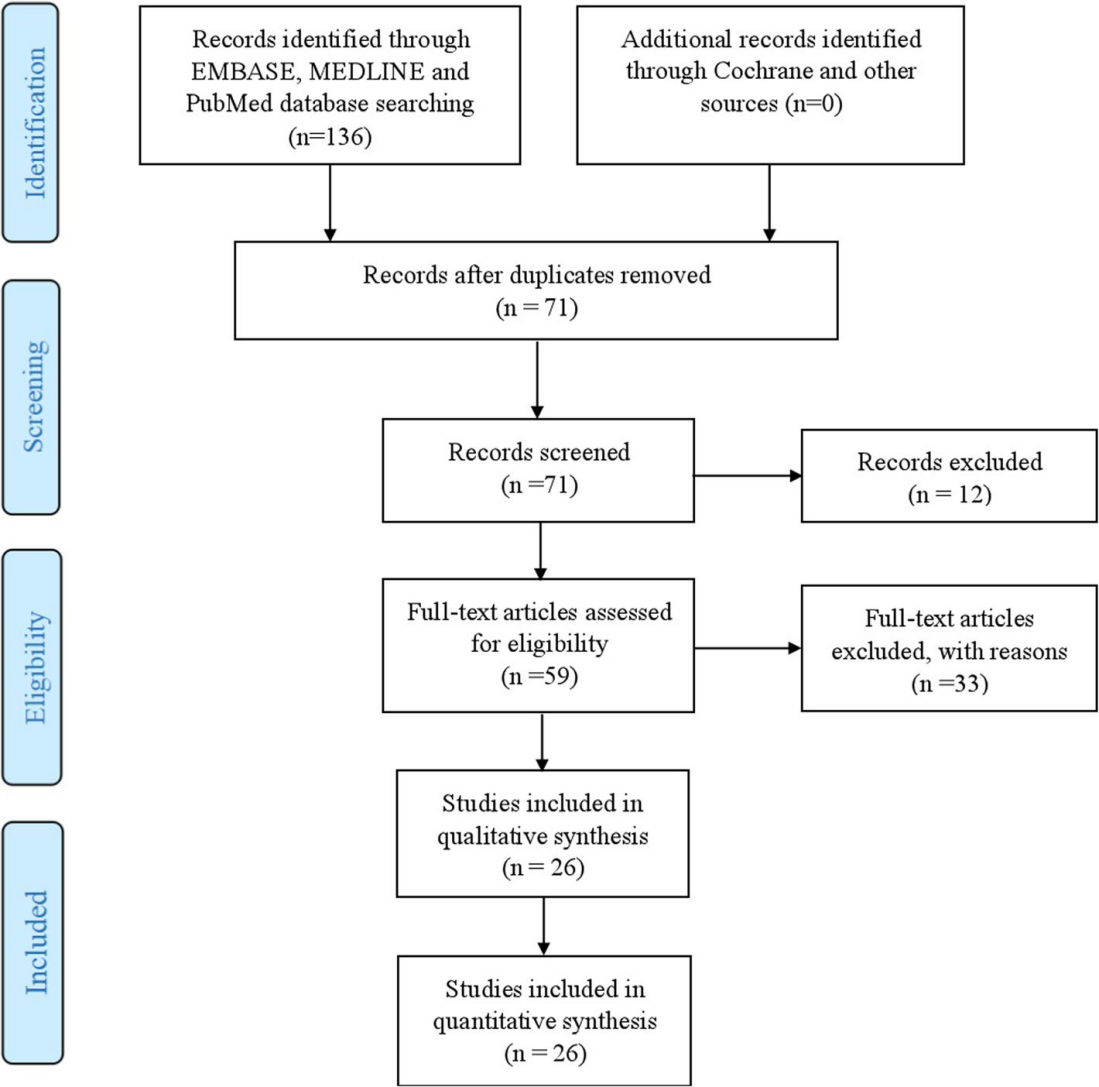
PRISMA flowchart

The combined searches yielded 136 abstracts, with 59 abstracts selected for full-text review. Of these 59, 33 studies were excluded because they reported ESESS/CSWSS/epilepsy-aphasia spectrum without underlying genetic etiologies or non-original data. 26 studies out of the 136 identified studies satisfied our inclusion criteria. This is summarized in PRISMA flowchart (Fig. 1). A total number of 151 cases were identified in those 26 studies. 124 cases were diagnosed with ESESS/CSWSS/epilepsy-aphasia spectrum solely.

### Monogenic mutations which associate with ESESS/CSWSS/epilepsy-aphasia spectrum

We identified 11 monogenic mutations which have been reported to associate with ESESS/CSWSS/epilepsy-aphasia spectrum. These included: *SCN2A* (N = 6), *NHE6*/*SLC9A6* (N = 1), *DRPLA*/*ATN1* (N = 1), Neuroserpin/*SRPX2* (N = 1), *OPA3* (N = 1), *KCNQ2* (N = 2)*, KCNA2* (N = 5), *GRIN2A* (N = 34), *CNKSR2* (N = 2), *SLC6A1* (N = 2) and *KCNB1* (N = 5). Seven genes were noticed in ESESS/CSWSS/epilepsy-aphasia spectrum solely including *SCN2A, KCNQ2, KCNA2, GRIN2A, CNKSR2, SLC6A1* and *KCNB1*. The underlying pathway for the *SCN2A, KCNQ2, KCNB1*, *KCNA2* and *GRIN2A* was channelopathy (N = 52). Four genes were noticed in patients with certain syndromes (*NHE6/SLC9A6*, *DRPLA/ ATN1*, Neuroserpin/*SRPX2,* and *OPA3*). Those syndromes include Christianson syndrome, Dentatorubro-pallidoluysian atrophy, Familial encephalopathy with neuroserpin inclusion bodies, and Costeff syndrome. Table 1 summarizes this information.

**Table 1:** Reported monogenic mutations which associate with ESESS/CSWSS/epilepsy aphasia spectrum

Gene	Location	OMIM number	Protein	Mutations	Number of reported cases	Spike-Wave Index	Associated syndromes or diagnosis	Underlying pathway	Author and date
*SCN2A*	2q24.3	182,390	Voltage-gated sodium channel Nav1.2	Loss of function.	6	Unknown	ESESS/CSWSS	Channelopathy	Wolff M et al. 2017 [21]
*NHE6/SLC9A6*	Xq26.3	300,231	Solute carrier family 9, subfamily A member 6	*De novo* splice site mutation (IVS10-1G > A)	1	> 85%	Christianson syndrome.	Na+/H+ exchanger	Zanni G et al. 2014 [11]
*DRPLA/ ATN1*	12p13.31	607,462	Atrophin 1	Expansion of the CAG repeat.	1	41.4%	Dentatorubro-pallidoluysian atrophy	Transcriptional co-repressor.	Kobayashi K et al. 2006 [22]
*Neuroserpin/ SRPX2*	Xq22.1	300,642	Sushi repeat containing protein, X-linked 2	*De novo* G392R mutation.	1	Unknown	Familial encepha-lopathy with neuroserpin inclusion bodies	Angiogenesis, Synaptogenesis	Coutelier M et al. 2008 [23]
*KCNQ2*	20q13.33	602,235	Potassium voltage-gated channel subfamily Q member 3	Deletion at E515D p	2	> 50%	ESESS/CSWSS	Channelopathy	Lee IC et al. 2017 [24]
*OPA3*	19q13.32	165,300	Outer mitochondrial membrane lipid metabolism regulator	(c.143-1G > C)	1	85%	Costeff syndrome	Regulates the shape of mitochondria.	Carmi N et al. 2015 [14]
*KCNA2*	1p13.3	176,262	Potassium voltage-gated channel subfamily A member 2	*De novo* c.1214 C > T (p.Pro405Leu) mutation	1	> 90%	ESESS/CSWSS	Channelopathy	Sachdev M et al. 2017 [25]
*KCNA2*	1p13.3	176,262	Potassium voltage-gated channel subfamily A member 2	*De novo* c.1214C4T p.Pro405Leu	1	> 80%	ESESS/CSWSS	Channelopathy	Syrbe S et al. 2015 [26]
*KCNA2*	1p13.3	176,262	Potassium voltage-gated channel subfamily A member 2	*De novo* c.1214C4T p.Pro405Leu	1	80–100%	ESESS/CSWSS	Channelopathy	Syrbe S et al. 2015 [26]
*KCNA2*	1p13.3	176,262	Potassium voltage-gated channel subfamily A member 2	*De novo* c.1214C4T p.Pro405Leu	1	70–75%	ESESS/CSWSS	Channelopathy	Syrbe S et al. 2015 [26]
*KCNA2*	1p13.3	176,262	Potassium voltage-gated channel subfamily A member 2	c.1214C4T p.Pro405Leu	1	> 70%	ESESS/CSWSS	Channelopathy	Masnada S et al. 2017 [27]
*GRIN2A*	16p13.2	138,253	Glutamate ionotropic receptor NMDA type subunit 2A	*De novo* c.2191G > A (p.Asp731Asn)	1	80%	ESESS/CSWSS/epilepsy aphasia	Channelopathy	Gao K et al. 2017 [28]
*GRIN2A*	16p13.2	138,253	Glutamate ionotropic receptor NMDA type subunit 2A	c.1123–2A > G	1	> 50%	ESESS/CSWSS/ epilepsy aphasia	Channelopathy	Lesca G et al. 2013 [29]
*GRIN2A*	16p13.2	138,253	Glutamate ionotropic receptor NMDA type subunit 2A	c.4161C > A	1	> 50%	ESESS/CSWSS	Channelopathy	Lesca G et al. 2013 [29]
*GRIN2A*	16p13.2	138,253	Glutamate ionotropic receptor NMDA type subunit 2A	Deletion at c.1510C > T	1	> 50%	LKS	Channelopathy	Lesca G et al. 2013 [29]
*GRIN2A*	16p13.2	138,253	Glutamate ionotropic receptor NMDA type subunit 2A	Deletion at c.1447G > A	1	> 50%	ESESS/CSWSS/ epilepsy aphasia	Channelopathy	Lesca G et al. 2013 [29]
*GRIN2A*	16p13.2	138,253	Glutamate ionotropic receptor NMDA type subunit 2A	Deletion at c.1553G > A	1	> 50%	ESESS/CSWSS/ epilepsy aphasia	Channelopathy	Lesca G et al. 2013 [29]
*GRIN2A*	16p13.2	138,253	Glutamate ionotropic receptor NMDA type subunit 2A	Deletion at c.2191G > A	1	> 50%	ESESS/CSWSS/ epilepsy aphasia	Channelopathy	Lesca G et al. 2013 [29]
*GRIN2A*	16p13.2	138,253	Glutamate ionotropic receptor NMDA type subunit 2A	Deletion at c.3751G > A	1	> 50%	ESESS/CSWSS/ epilepsy aphasia	Channelopathy	Lesca G et al. 2013 [29]
*GRIN2A*	16p13.2	138,253	Glutamate ionotropic receptor NMDA type subunit 2A	Deletion at c.2146G > A	1	> 50%	ESESS/CSWSS/ epilepsy aphasia	Channelopathy	Lesca G et al. 2013 [29]
*GRIN2A*	16p13.2	138,253	Glutamate ionotropic receptor NMDA type subunit 2A	Deletion at c.2797G > A	1	> 50%	LKS	Channelopathy	Lesca G et al. 2013 [29]
*GRIN2A*	16p13.2	138,253	Glutamate ionotropic receptor NMDA type subunit 2A	Deletion at c.551 T > G	1	> 50%	ESESS/CSWSS	Channelopathy	Lesca G et al. 2013 [29]
*GRIN2A*	16p13.2	138,253	Glutamate ionotropic receptor NMDA type subunit 2A	*De novo* deletion at c.2081 T > C	1	> 50%	LKS	Channelopathy	Lesca G et al. 2013 [29]
*GRIN2A*	16p13.2	138,253	Glutamate ionotropic receptor NMDA type subunit 2A	*De novo* deletion at c.1954 T > G	1	> 50%	ESESS/CSWSS	Channelopathy	Lesca G et al. 2013 [29]
*GRIN2A*	16p13.2	138,253	Glutamate ionotropic receptor NMDA type subunit 2A	*De novo* deletion at c.1642G > A	1	> 50%	LKS	Channelopathy	Lesca G et al. 2013 [29]
*GRIN2A*	16p13.2	138,253	Glutamate ionotropic receptor NMDA type subunit 2A	Deletion at c.2007G > T	1	> 50%	ESESS/CSWSS/ epilepsy aphasia	Channelopathy	Lesca G et al. 2013 [29]
*GRIN2A*	16p13.2	138,253	Glutamate ionotropic receptor NMDA type subunit 2A	Deletion at c.883G > A	1	> 50%	ESESS/CSWSS	Channelopathy	Lesca G et al. 2013 [29]
*GRIN2A*	16p13.2	138,253	Glutamate ionotropic receptor NMDA type subunit 2A	c.3827C > G	1	> 50%	ESESS/CSWSS	Channelopathy	Lesca G et al. 2013 [29]
*GRIN2A*	16p13.2	138,253	Glutamate ionotropic receptor NMDA type subunit 2A	c.1005-1C > T	1	> 85%	ESESS/CSWSS/ epilepsy aphasia	Channelopathy	Carvill GL et al. 2013 [30]
*GRIN2A*	16p13.2	138,253	Glutamate ionotropic receptor NMDA type subunit 2A	c.2A > G	1	> 85%	ESESS/CSWSS/ epilepsy aphasia	Channelopathy	Carvill GL et al. 2013 [30]
*GRIN2A*	16p13.2	138,253	Glutamate ionotropic receptor NMDA type subunit 2A	c.1005-1C > T	1	> 85%	ESESS/CSWSS/ epilepsy aphasia	Channelopathy	Carvill GL et al. 2013 [30]
*GRIN2A*	16p13.2	138,253	Glutamate ionotropic receptor NMDA type subunit 2A	c.1592G > A	1	> 85%	ESESS/CSWSS/ epilepsy aphasia	Channelopathy	Carvill GL et al. 2013 [30]
*GRIN2A*	16p13.2	138,253	Glutamate ionotropic receptor NMDA type subunit 2A	c.2041C > T	1	> 85%	LKS	Channelopathy	Lemke JR et al. 2013 [31]
*GRIN2A*	16p13.2	138,253	Glutamate ionotropic receptor NMDA type subunit 2A	c.1007 + 1G > A	1	> 85%	LKS	Channelopathy	Lemke JR et al. 2013 [31]
*GRIN2A*	16p13.2	138,253	Glutamate ionotropic receptor NMDA type subunit 2A	c.2140G > A	1	> 85%	ESESS/CSWSS	Channelopathy	Lemke JR et al. 2013 [31]
*GRIN2A*	16p13.2	138,253	Glutamate ionotropic receptor NMDA type subunit 2A	c.2927A > G	1	> 85%	ESESS/CSWSS	Channelopathy	Lemke JR et al. 2013 [31]
*GRIN2A*	16p13.2	138,253	Glutamate ionotropic receptor NMDA type subunit 2A	c.1001 T > A	1	> 85%	ESESS/CSWSS	Channelopathy	Lemke JR et al. 2013 [31]
*GRIN2A*	16p13.2	138,253	Glutamate ionotropic receptor NMDA type subunit 2A	c.2334_2338delCTTGC	1	> 85%	ESESS/CSWSS	Channelopathy	Lemke JR et al. 2013 [31]
*GRIN2A*	16p13.2	138,253	Glutamate ionotropic receptor NMDA type subunit 2A	c.2829C > G	1	> 85%	ESESS/CSWSS	Channelopathy	Lemke JR et al. 2013 [31]
*GRIN2A*	16p13.2	138,253	Glutamate ionotropic receptor NMDA type subunit 2A	c.2007 + 1G > A	1	> 85%	ESESS/CSWSS	Channelopathy	Lemke JR et al. 2013 [31]
*GRIN2A*	16p13.2	138,253	Glutamate ionotropic receptor NMDA type subunit 2A	c.236C > G	1	> 85%	ESESS/CSWSS	Channelopathy	Lemke JR et al. 2013 [31]
*GRIN2A*	16p13.2	138,253	Glutamate ionotropic receptor NMDA type subunit 2A	c.692G > A	1	> 85%	LKS	Channelopathy	Lemke JR et al. 2013 [31]
*GRIN2A*	16p13.2	138,253	Glutamate ionotropic receptor NMDA type subunit 2A	c.1637_1639delCTT	1	> 85%	ESESS/CSWSS	Channelopathy	Lemke JR et al. 2013 [31]
*GRIN2A*	16p13.2	138,253	Glutamate ionotropic receptor NMDA type subunit 2A	c.1007 + 1G > A	1	> 85%	LKS	Channelopathy	Lemke JR et al. 2013 [31]
*GRIN2A*	16p13.2	138,253	Glutamate ionotropic receptor NMDA type subunit 2A	c.1007 + 1G > A	1	> 85%	ESESS/CSWSS	Channelopathy	Lemke JR et al. 2013 [31]
*CNKSR2*	Xp22.12	300,724	Connector enhancer of KSR2	Novel nonsense mutation (c.2314 C > T; p.Arg712*)	2	Unknown	ESESS/CSWSS	Synaptic protein	Damiano JA et al. 2017 [32]
*SLC6A1*	3p25.3	137,165	Solute carrier family 6 member 1	*De novo* c.695G > T, p.Gly232Val	1	82%	ESESS/CSWSS	Gamma-aminobutyric acid (GABA) transporter	Johannesen KM et al. 2018 [33]
*SLC6A1*	3p25.3	137,165	Solute carrier family 6 member 1	*De novo* c.1369_1370 delGG Gly457HisFsTer10	1	Almost continuous epileptic activity.	ESESS/CSWSS	Gamma-aminobutyric acid (GABA) transporter	Johannesen KM et al. 2018 [33]
*KCNB1*	20q13.13	616,056	Potassium channel, voltage-gated, shab-related subfamily, member 1	chr20:47990976G > Ap.T374I, chr20:47991162C > Tp.R312H, chr20:47991162C > Tp.R312H, chr20:47991181G > Ap.R306C, Chr20:47991468G > Tp.T210 K	5	Unknown	ESESS/CSWSS	Channelopathy	de Kovel CGF, et al. 2017 [34]

OMIM; Online Mendelian Inheritance in Man, ESESS; electrical status epilepticus during slow-wave sleep, CSWSS; continuous spike-wave of slow sleep

### Copy number variations which associate with ESESS/CSWSS/epilepsy aphasia spectrum

We identified 89 CNVs which have been reported to associate with ESESS/CSWSS/epilepsy-aphasia spectrum: 9 recurrent and 45 non recurrent. Recurrent CNVs included 15q11.2–13.1 duplication for 15 cases, 3q29 duplication for 11 cases, Xp22.12 deletion for 4 cases, 16p13 deletion for 4 cases, 11p13 duplication for 2 cases, 10q21.3 deletion for 2 cases, 3q25 deletion for 2 cases, 8p23.3 deletion for 2 cases and 9p24.2 duplication for 2 cases. 61 CNVs were noticed in patients with ESESS/CSWSS/epilepsy-aphasia spectrum solely. 4 of the 61 CNVs spanned gene involved in channel *(GRIN2A*)*,* 8 CNVs spanned genes involved in synaptic connection (*CNKSR2*, *SHANK3* and *DLG2*), and 14 CNVs spanned genes involved in cell adhesion (*CDH13, CTNNA3, DIAPH3, CDH9, CDH6, KIF26B, CDH4, CNTNAP2, SGCZ, HIPK3, CSTF3* and *CNTN6*). Three CNVs were noticed in patients with certain syndromes including, 8p deletion syndrome and 9p duplication syndrome. Table 2 summarizes this information.

**Table 2:** Reported pathogenic copy number variations which associate with ESESS/CSWSS/epilepsy-aphasia spectrum

Chromosomal location	Coordinates	Type of aberration	Size	Number genes	Gene associated with ESESS/CSWSS	Number of cases	Associated syndromes or diagnosis	Spike-Wave Index	Underlying pathway	Author and date
8q12.3q13.2	Chr8:65,236,018–68,801,113	Del	3.57 Mb	27	Unknown	1	ESESS/CSWSS	60–70%	Unknown	Willem M.A et al. 2011 [52]
Xp11.22–11.23.	Unknown	Dup	0.8–9.2 Mb.		Unknown	5	Microduplication syndrome	Unknown	Unknown	Giorda R et al. 2009 [53]
16p13	Chr16:10,246,239–10,354,862	Del	109Kb	1	*GRIN2A*	4	ESESS/CSWSS	> 50%	Channelopathy	Lesca et al., 2012 [12], Constanze Reutlinger et al. 2010 [54]
8p23.3	Unknown	Del	1.8-Mb	3	*FBXO25*	2	8p deletion syndrome	20–30%	Unknown	Tojo Nakayama et al. 2012 [55]
9p24.2	Unknown	Dup	2.6-Mb		Unknown	2	9p duplication syndrome	50–60%	Unknown	Tojo Nakayama et al. 2012 [55]
14q21.3	Chr14:47,592,578–47,662,394	Del	70Kb	1	*MDGA2*	1	ESESS/CSWSS	> 50%	Metabolism of proteins	Lesca et al. 2012 [12]
22q13.32-q13.33	Chr22:49,346,697–51,219,009	Del	1.9 Mb	45	*SHANK3*	1	ESESS/CSWSS	> 50%	Synapse connection	Lesca et al. 2012 [12]
16q23.3	Chr16:83,599,498–83,857,382	Del	258Kb	2	*CDH13*	1	ESESS/CSWSS	> 50%	Cell adhesion	Lesca et al. 2012 [12]
15q13.3	Chr15:29,000,000–30,400,000	Del	1.4 Mb	7	*CHRNA7*	1	LKS	> 75%	Cholinergic pathway	Kevelam SH et al. 2012 [20]
Xp22.11	ChrX:24,270,000–24,760,000	Dup	490Kb	3	*PCYT1B*	1	ESESS/CSWSS	> 75%	Cholinergic pathway	Kevelam SH et al. 2012 [20]
5p12	Chr5:43,820,000–44,380,000	Dup	560Kb	1	*FGF10*	1	ESESS/CSWSS	> 75%	Growth factor activity.	Kevelam SH et al. 2012 [20]
5q31.3	Chr5: 141,970,000–142,430,000	Dup	560Kb	2	*FGF1, ARHGAP26*	1	ESESS/CSWSS	> 75%	Cell growth	Kevelam SH et al. 2012 [20]
16q23.1	Chr16: 75,750,000–76,220,000	Dup	470Kb	2	*ADAMTS18, MON1B*	1	ESESS/CSWSS	> 75%	Metabolism of proteins	Kevelam SH et al. 2012 [20]
9q34.3	Chr9: 138,150,000–138,380,000	Dup	230Kb	5	*LHX, QSOX2, GPSM1, CORF151, CARD9*	1	ESESS/CSWSS	> 75%	Cell apoptosis	Kevelam SH et al. 2012 [20]
15q11.2–13.1	Unknown	Dup	Unknown		Unknown	15	ESESS/CSWSS	50%	Unknown	Arkilo D et al. 2016 [56]
10q21.3	Chr10:68,438,375–68,506,557	Del	68 Kb	1	*CTNNA3*	1	ESESS/CSWSS	> 50%	Cell adhesion	Lesca G et al. 2012 [12]
13q21.2	Chr13:60,419,603–60,647,521	Del	228 Kb	1	*DIAPH3*	1	ESESS/CSWSS	> 50%	Cell adhesion	Lesca G et al. 2012 [12]
5p14.1	Chr5:28,634,980–28,837,425	Del	202 Kb	2	*CDH9* and *CDH6*	1	ESESS/CSWSS	> 50%	Cell adhesion	Lesca G et al., 2012 [12]
10q21.3	Chr10:68,251,535–68,496,866	Del	245 Kb	1	*CTNNA3*	1	ESESS/CSWSS	> 50%	Cell adhesion	Lesca G et al. 2012 [12]
10q21.3	Chr10:68,550,481–68,668,009	Del	118 Kb	1	*CTNNA3*	1	ESESS/CSWSS	> 50%	Cell adhesion	Lesca G et al. 2012 [12]
20p12.1	Chr20:14,491,297–14,591,133	Del	100 Kb	1	*MACROD2*	1	ESESS/CSWSS	> 50%	Deacetylates O-acetyl-ADP ribose.	Lesca G et al. 2012 [12]
1q44	Chr1:245,320,978–245,410,054	Dup	89 Kb	1	*KIF26B*	1	LKS-ESESS/CSWSS	> 50%	Cell adhesion	Lesca G et al. 2012 [12]
3q28-q29	Chr3:192,067,520–192,122,231	Dup	54 Kb	1	*FGF12*	1	ESESS/CSWSS	> 50%	Growth factor activity and ion channel binding.	Lesca G et al. 2012 [12]
3q29	Chr3:192,212,953–192,352,465	Dup	139 Kb	1	*FGF12*	1	ESESS/CSWSS	> 50%	Growth factor activity and ion channel binding.	Lesca G et al. 2012 [12]
3q29	Chr3:192,870,621–193,385,022	Dup	514 Kb	5	*HRASLS, ATP13A5, ATP* *13A4, OPA1*	5	ESESS/CSWSS	> 50%	Metabolism	Lesca G et al. 2012 [12]
20p12.1	Chr20:14,395,797–14,464,507	Dup	29 Kb	1	*MACROD2*	1	LKS-ESESS/CSWSS	> 50%	Deacetylates O-acetyl-ADP ribose.	Lesca G et al. 2012 [12]
20q13.3	Chr20:60,015,337–60,078,775	Del	63 Kb	1	*CDH4*	1	LKS-ESESS/CSWSS	> 50%	Cell adhesion	Lesca G et al. 2012 [12]
10q21.3	Chr10:68,087,319–68,110,043	Del	23 Kb	1	*CTNNA3*	1	LKS-ESESS/CSWSS	> 50%	Cell adhesion	Lesca G et al. 2012 [12]
7q35	Chr7:146,226,258–146,254,837	Dup	29 Kb	1	*CNTNAP2*	1	LKS-ESESS/CSWSS	> 50%	Cell adhesion	Lesca G et al. 2012 [12]
8p23.1	Chr8:9,598,226–10,787,792	Del	1,189Kb	8	*TNKS, MIR124–1,MSRA, PRSS55, RP1L1, SOX7, PINX1, XKR6*	1	ESESS/CSWSS	> 50%	Cell growth	Lesca G et al. 2012 [12]
8q21	Chr8:89,102,084–89,398,298	Del	296Kb	1	*MMP16*	1	ESESS/CSWSS	> 50%	Breakdown of extracellular matrix	Lesca G et al. 2012 [12]
1q25.3	Chr1:183,594,532–183,820,790	Dup	226Kb	3	*ARPC5, APOBEC4, RGL1*	1	ESESS/CSWSS	> 50%	Gene expression	Lesca G et al. 2012 [12]
3q25	Chr3:158,183,313–158,296,641	Del	113Kb	3	*RSRC1* and *MLF1*	1	ESESS/CSWSS	> 50%	Pre-mRNA splicing	Lesca G et al. 2012 [12]
3q26.32–33	Chr3:178,969,064–179,150,965	Dup	182Kb	4	*KCNMB3, ZNF639, MFN1, GNB4*	1	ESESS/CSWSS	> 50%	Potassium and Calcium channel regulator	Lesca G et al. 2012 [12]
Xp22.12	ChrX:21,523,673–21,558,329	Del	35Kb	1	*CNKSR2*	1	ESESS/CSWSS	> 50%	Synaptic proteins	Lesca G et al. 2012 [12]
Xp22.12	ChrX:21,328,677–21,670,497	Del	342 Kb	1	*CNKSR2*	1	ESESS/CSWSS	Frequent and nearly continuous independent discharges.	Synaptic proteins	Aypar U et al. 2015 [47]
Xp22.12	ChrX:21,285,233–21,519,405	Del	234 Kb	1	*CNKSR2*	1	ESESS/CSWSS/epilepsy aphasia	Unknown	Synaptic proteins	Houge G et al. 2012 [48]
Xp22.12	ChrX:20,297,696–21,471,387	Del	1.17 Mb	1	*CNKSR2*	1	ESESS/CSWSS	80 to 100%	Synaptic proteins	Vaags AK et al. 2014 [49]
Xp22.12	ChrX:20,297,696–21,471,387	Del	1.17 Mb	1	*CNKSR2*	1	ESESS/CSWSS	> 80%	Synaptic proteins	Vaags AK et al. 2014 [49]
Xp22.12	ChrX:21,375,312–21,609,484	Del	234 Kb	1	*CNKSR2*	1	ESESS/CSWSS	> 80%	Synaptic proteins	Vaags AK et al. 2014 [49]
3q25	Chr3:154,395,454–154,788,305	Del	393Kb	1	*MME*	1	ESESS/CSWSS	> 50%	Peptidase activity and endopeptidase activity	Lesca G et al. 2012 [12]
5q11.2	Chr5:58,571,292–58,745,139	Del	174Kb	1	*PDE4D*	1	ESESS/CSWSS	> 50%	Protein domain specific binding.	Lesca G et al. 2012 [12]
6q27	Chr6:167,355,901–167,373,534	Del	18Kb	1	*RNASET2*	1	LKS-ESESS/CSWSS	> 50%	RNA catabolism.	Lesca G et al. 2012 [12]
7q22	Chr7:107,214,193–107,262,539	Del	48Kb	2	*DUS4L* and *BCAP29*	1	ESESS/CSWSS	> 50%	Unknown	Lesca G et al. 2012 [12]
8p22	Chr8:14,553,553–14,572,370	Del	19Kb	1	*SGCZ*	1	ESESS/CSWSS	> 50%	Cell adhesion	Lesca G et al. 2012 [12]
8q22.3	Chr8:102,849,359–102,868,211	Del	19Kb	1	*NCALD*	1	ESESS/CSWSS	> 50%	Calcium binding protein	Lesca G et al. 2012 [12]
1p21.2–21.1	Chr1:102,123,099–103,099,662	Dup	977Kb	1	*OLMF3*	1	ESESS/CSWSS	> 50%	Unknown	Lesca G et al. 2012 [12]
3p11.2	Chr3:87,917,810–88,778,873	Dup	29 KB	4	*HTR1F, CGGBP1, ZNF654, C3orf38*	1	ESESS/CSWSS	> 50%	Serotonin receptor	Lesca G et al. 2012 [12]
3q29	Chr3:194,088,557–194,130,145	Dup	217Kb	5	*LRRC15, GP5, ATP13A3, LOC100131551*	1	ESESS/CSWSS	> 50%	Transportation of cations	Lesca G et al. 2012 [12]
8q11.23	Chr8:53,397,126–53,808,953	Dup	412Kb	2	*FAM150A, RB1CC1*	1	LKS-ESESS/CSWSS	> 50%	Regulation of neuronal homeostasis	Lesca G et al. 2012 [12]
9p13.2	Chr9:37,299,058–37,451,697	Dup	153Kb	3	*ZCCHC7, GRHPR, ZBTB5*	1	LKS-ESESS/CSWSS	> 50%	Metabolism	Lesca G et al. 2012 [12]
10q21.1	Chr10:56,034,426–56,089,442	Dup	55Kb	1	*PCDH15*	1	ESESS/CSWSS	> 50%	Protocadherin	Lesca G et al. 2012 [12]
14q21.3	Chr14:46,524,008–47,161,263	Dup	637Kb	1	*RPL10L*	1	ESESS/CSWSS	> 50%	Unknown	Lesca G et al. 2012 [12]
8p23.2	Chr8:4,289,484–4,388,709	Del	99Kb	1	*CSMD1*	1	LKS-ESESS/CSWSS	> 50%	Unknown	Lesca G et al. 2012 [12]
11p13	Chr11:33,179,961–33,339,337	Dup	159Kb	2	*HIPK3, CSTF3*	1	LKS-ESESS/CSWSS	> 50%	Cell adhesion	Lesca G et al. 2012 [12]
11p13	Chr11:33,249,368–33,349,707	Dup	100Kb	1	*HIPK3*	1	LKS-ESESS/CSWSS	> 50%	Cell adhesion	Lesca G et al. 2012 [12]
11p15.5	Chr11:1,468,991–1,491,145	Dup	22Kb	1	*BRSK2*	1	ESESS/CSWSS	> 50%	Regulates polarization of neurons	Lesca G et al. 2012 [7]
10q21.1	Chr10:56,626,171–56,691,361	Del	65Kb	1	*PCDH15*	1	ESESS/CSWSS	> 50%	Protocadherin	Lesca G et al. 2012 [7]
11q14	Chr11:84,539,606–84,565,141	Del	26Kb	1	*DLG2*	1	LKS-ESESS/CSWSS	> 50%	Synaptic transmission	Lesca G et al. 2012 [7]
Xq28	ChrX:154,396,991–154,425,684	Del	29Kb	1	*AK301646*	1	ESESS/CSWSS	> 50%	Unknown	Lesca G et al. 2012 [7]
Xp22.31	ChrX:6,489,877–8,131,810	Del	1642	5	*HDHD1, STS, VCX, PNPLA4, MIR651*	1	ESESS/CSWSS	> 50%	Phospholipases	Lesca G et al. 2012 [7]
2p21	Chr2:45,410,272–45,961,582	Dup	551Kb	3	*UNQ6975, SRBD1, PRKCE*	1	LKS-ESESS/CSWSS	> 50%	Reward signaling	Lesca G et al. 2012 [7]
Xp21.1	ChrX:30,615,032–30,888,295	Dup	273Kb	2	*GK, MAP3K7IP3*	1	ESESS/CSWSS	> 50%	Immune system	Lesca G et al. 2012 [12]
3p26.3	Chr3:1,414, 614–1,620,145	Dup	206Kb	1	*CNTN6*	1	ESESS/CSWSS	> 50%	Cell adhesion	Lesca G et al. 2012 [7]

ESESS; electrical status epilepticus during slow-wave sleep, CSWSS; continuous spike-wave of slow sleep, Del; deletion, Dup; duplication

## Discussion

Electrical status epilepticus during slow-wave sleep which is also known as continuous spike-wave of slow sleep is type of an EEG pattern which is seen in ESESS/CSWSS/epilepsy aphasia spectrum with an estimated prevalence of 0.5%. However, this prevalence might be inaccurate due to a few studies which have been done on it as well as the usage of different diagnostic criteria in making diagnosis. ESESS/CSWSS associates with long-term neuropsychological impairment. It can occur alone or with other syndromes. We aimed to review all reported genetic etiologies of ESESS/CSWSS/epilepsy-aphasia spectrum and to study their possible underlying pathway especially for ESESS/CSWSS/epilepsy aphasia spectrum which occurs alone. This review will provide an insight regarding the contribution of genetic etiologies in ESESS/CSWSS/epilepsy-aphasia spectrum and the possible common underlying pathway which can assist in identification of the appropriate therapy. Identification of the target therapy will help to reduce the long-term neuropsychological impairment.

We have observed that approximately 67.6% (*N* = 102/151) of the cases were diagnosed with ESESS/CSWSS/epilepsy-aphasia spectrum when they had spike-wave index > 50% clearly activated during sleep while 13.2% (*N* = 20/151) were diagnosed when they had spike-wave index > 85%. Our finding differs from the survey which was done by Fernández IS et al. in North America where they found 57.6% of the members of the Child Neurology Society and the American Epilepsy Society considered a cut-off value of 85% spike-wave index while 30.8% considered a cut-off value of 50% [4]. This difference could be due to the fact that our review involved multiple studies from different areas of the world. Currently, there is no specific criteria from ILAE for definition of ESESS/CSWSS pattern hence jeopardize communication among clinicians and research in this condition. We suggest development of common cut-off value.

A total number of 11 monogenic mutations and 89 CNVs were identified to associate with ESESS/CSWSS/epilepsy-aphasia spectrum. Monogenic mutations included *SCN2A* [21], *NHE6*/S*LC9A6* [11], *DRPLA*/*ATN1* [22], Neuroserpin/*SRPX2* [23], *KCNQ2* [24], *OPA3* [14], *KCNA2* [25–27], *GRIN2A* [28–31]*, CNKSR2* [32], *SLC6A1* [33] and *KCNB1* [34]*.* Seven genes were noticed in ESESS/CSWSS/epilepsy-aphasia spectrum solely including *SCN2A, KCNQ2, KCNA2, GRIN2A, CNKSR2, SLC6A1* and *KCNB1.* Out of 89 CNVs, 9 were recurrent whilst 45 were non-recurrent. 4 CNVs spanned gene involved in channel *(GRIN2A*)*,* 8 CNVs spanned genes involved in synaptic connection (*CNKSR2*, *SHANK3* and *DLG2*), and 14 CNVs spanned genes involved in cell adhesion (*CDH13, CTNNA3, DIAPH3, CDH9, CDH6, KIF26B, CDH4, CNTNAP2, SGCZ, HIPK3, CSTF3* and *CNTN6).* 68 of the reported genetic etiologies including monogenic mutations and CNVs were detected in patients with ESESS/CSWSS/epilepsy-aphasia spectrum solely. The most common underlying pathway was channelopathy (N = 56). The pathogenic genes included *SCN2A*, *KCNQ2*, *KCNB1*, *KCNA2* and *GRIN2A* (Tables 1 and 2**)**.

*SCN2A* gene encodes subunits of voltage-gated sodium channel which is responsible for generation and propagation of action potentials in neurons and muscles [35]. *SCN2A* mutations associate with two phenotypic spectrum related to epilepsy: the early onset (< 3 months) group which include benign familial neonatal or infantile seizures (BFNIS) and the late onset (> 3 months) group which include focal epilepsies with an ESESS/CSWSS-like picture [21]. Wolff M et al. studied the phenotypes of cases with *SCN2A* mutations in which they discovered three patients with A263V mutation who showed BFNIS phenotype, while three others with the same mutation had more severe phenotypes [21]. They concluded that, both the mutation itself and other genetic or environmental factors contribute to the individual phenotype. We identified only 6 patients with ESESS/CSWSS/epilepsy-aphasia spectrum who were reported to have *SCN2A* mutations. The few cases could be explained by other unknown genetic or environmental factors which could contribute to the phenotype. Additionally, it could be due to a few studies that have focused on identification of genetic etiologies in this particular condition.

*KCNQ2* gene encodes for subunits of potassium channel which is highly expressed in brain neurons [36]. It produce M- current which prevents constant neuronal excitability and hence prevent seizures. *KCNQ2* mutations associate with wide range of phenotypes: BFNIS, benign familial infantile seizures, neonatal onset epileptic encephalopathies, and ESESS/CSWSS. The wide range of phenotypes depend on the position and the features of the amino acid change which result to variable voltage sensitivity of the channel [37–40]. The change can promote increase and decrease of channel activity leading to different intensity levels hence different phenotypes. Other researchers have hypothesized that different phenotypes could be explained by an interplay of pathogenic mutations, modifier genes, and other environmental factors [41]. We identified only two reported cases with ESESS/CSWSS/epilepsy-aphasia spectrum who had *KCNQ2* mutations [24]. The few cases could be explained by the kind of mutation, and other unknown modifier genes and environmental factors.

*KCNA2* gene encodes potassium channel, voltage-gated, shaker-related subfamily and is highly expressed in brain and central nervous system [42]. It prevents abnormal action potential firing and regulates neuronal output. *KCNA2* mutations associate with two types of phenotypes based on the severity of the encephalopathy and of the seizure disorder. The milder phenotypes correlates with loss-of-function mutations and more severe phenotypes with gain-of-function mutations [26]. Sachdev M et al. [25], Syrbe S et al [26] and Masnada S et al [27] reported a total number of five patients who were diagnosed with ESESS/CSWSS/epilepsy- aphasia spectrum and found to have *KCNA2* mutations.

*GRIN2A* gene encodes N-methyl-D-aspartate (NMDA) glutamate receptor α2 subunit [43]. The NMDA receptor is a glutamate-activated ion channel permeable to sodium, potassium and calcium and is found at excitatory synapses throughout the brain. The current which is produced by NMDA receptor–mediated component of the excitation is crucial in the central nervous system as it determines the key features of the synaptic response and has important consequences for synaptic plasticity and cell physiology. Dysfunction of NMDA receptor–mediated signaling can trigger and/or influence numerous brain diseases, including epilepsy [44]. Four studies have reported 34 patients with *GRIN2A* mutations who were diagnosed with ESESS/CSWSS/epilepsy-aphasia spectrum [28–31]. Additionally, four patients with deletion at 16p13 spanning *GRIN2A* gene have been reported [12]. Miyamoto H et al. explained the relationship between NMDA receptor functioning and the modulation of ESESS/CSWSS [45]. Therefore, NMDA receptor can stand as a target for development of drug since *GRIN2A* mutations were reported in many patients with ESESS/CSWSS/epilepsy-aphasia spectrum (N = 38).

*KCNB1* gene encodes a member of the potassium channel, voltage-gated, shab-related subfamily. It is highly expressed in brain neurons [46]. *KCNB1* gene mutations associate with early infantile epileptic encephalopathies. de Kovel CGF et al. studied the phenotypes of 26 cases with *KCNB1* gene mutations in which 5 of them were identified to have ESESS/CSWSS [34].

*CNKSR2* gene encodes connector enhancer of KSR2 which is a synaptic protein involved in Ras signaling-mediated neuronal proliferation, migration and differentiation. Synaptic proteins are crucial for neuronal function in the brain, and their deficiency can lead to epilepsy and cognitive impairments. Damiano JA et al. reported a novel nonsense mutation (c.2314 C > T; p.Arg712*) in 2 siblings diagnosed with ESESS/CSWSS/epilepsy-aphasia spectrum [32]. Moreover, approximately 4 studies reported 6 patients with deletion at Xp22.12 spanning *CNKSR2* gene [12, 47–49]. Hence *CNKSR2* gene has a role to play in ESESS/CSWSS/epilepsy-aphasia spectrum.

*SLC6A1* gene encodes voltage-dependent gamma-aminobutyric acid (GABA) transporter 1 (GAT-1), one of the main GABA transporters in central nervous system [50]. The dysfunction of this transporter leads to spontaneous spike-wave discharges and absence seizures [51]. Johannesen KM et al. reviewed the phenotypic spectrum of 34 cases with *SLC6A1* mutations in which they identified two patients who presented with ESESS/CSWSS/epilepsy-aphasia spectrum [33]. This new finding suggests the role of GABA in pathogenesis of ESESS/CSWSS/epilepsy-aphasia spectrum.

Most of the reported CNVs span genes involved in cell adhesion (N = 14): *CDH13, CTNNA3, DIAPH3, CDH9, CDH6, KIF26B, CDH4, CNTNAP2, SGCZ, HIPK3, CSTF3* and *CNTN6*. Hence we support Lesca G et al in suggesting that, these genes might explain the role of autoimmunity in the pathogenesis of ESESS/CSWSS/epilepsy-aphasia spectrum [12]. However, they have never been reported as monogenic mutation.

We have observed that *SCN2A*, *KCNQ2*, *KCNB1*, *KCNA2* and *GRIN2A* contributed to the etiology of many patients with ESESS/CSWSS solely (Tables 1 and 2). The common underlying functions of these genes is to encode important channels in brain neurons. Their disturbances lead to ESESS/CSWSS/epilepsy aphasia spectrum. Therefore we think channelopathy plays a major role in pathogenesis of ESESS/CSWSS/epilepsy aphasia spectrum.

Several syndromes have been reported to associate with ESESS/CSWSS pattern including Christianson syndrome, Dentatorubro-pallidoluysian atrophy, Familial encephalopathy with neuroserpin inclusion bodies, Rolandic Epilepsy, Costeff syndrome, Landau-Kleffner syndrome, 8p deletion syndrome and 9p duplication syndrome. However, they have the separate possible underlying pathway (Tables 1 and 2). Our review was limited due to existing discrepancy in terms of diagnostic criteria (spike wave index) which are being used. Hence there is no common language.

## Conclusion

Approximately 37% (56/151) of the reported cases diagnosed with ESESS/CSWSS/epilepsy-aphasia spectrum solely had pathogenic genes responsible for encoding channels in the brain neurons. Consequently, our review suggests channelopathy as a possible underlying cause which can be targeted for the development of appropriate therapy. However, this remains non-conclusive because less cytogenetic studies have been performed in this particular condition. We argue more research to be performed in patients who present with ESESS/CSWSS/epilepsy-aphasia spectrum solely so as discover more underlying causes which will facilitate in proper therapy identification. We also suggest development of diagnostic criteria (cut-off value for spike-wave index) which can be utilized worldwide to ensure common language among clinicians and researchers.

## Additional file

Additional file 1: Search strategies which were used in MEDLINE, EMBASE, PubMed and Cochrane review database. (DOCX 13 kb)

## Abbreviations

CNVs: copy number variations
CSWSS: continuous spike-wave of slow sleep
ESESS: Electrical status epilepticus during slow-wave sleep
OMIM: Online Mendelian Inheritance in Man
