# Supplementary material for: Genetic etiologies of the electrical status epilepticus during slow wave sleep: systematic review
Source: BMC Genet. 2018 Jul 6;19:40. doi: 10.1186/s12863-018-0628-5 (PMC6034250; doi:10.1186/s12863-018-0628-5)
Supplement: Supplementary file 1 — Search strategies which were used in MEDLINE, EMBASE, PubMed and Cochrane review database. (DOCX 13 kb) [file 12863_2018_628_MOESM1_ESM.docx]

**Additional file 1.**

**Search strategies which were used.**

**EMBASE - Search strategy**

1. (electrical AND status AND epilepticus AND during AND slow AND ('wave'/exp OR wave) AND ('sleep'/exp OR sleep) OR as) AND continuous AND ('spike wave'/exp OR 'spike wave') AND of AND slow AND ('sleep'/exp OR sleep) AND ('genetics'/exp OR genetics) AND [1966-2017]/py.

**MEDLINE- Search strategy**

1. (TOPIC: (elecrical) AND (TOPIC: (status) OR MeSH HEADING:exp: (Social Environment) OR (MeSH HEADING:exp: ((Social Class)) OR MeSH HEADING:exp: ((Social Environment))))TOPIC: (epilepticus) OR TOPIC: (during) OR TOPIC: (sleep)) OR TOPIC: ((continuous) spikes AND waves during slow sleep) AND genetics) Timespan: All years. Indexes: MEDLINE. Timespan: All years. Search language=Auto.

**PubMed and Cochrane data base- search strategy.**

1. MeSH terms: sleep; status epilepticus; genetics; electricity

Subheadings: Genetics.

1. ("epilepsy"[MeSH Terms] OR "epilepsy"[All Fields] OR "epileptic"[All Fields]) AND ("aphasia"[MeSH Terms] OR "aphasia"[All Fields]) AND ("genetics"[Subheading] OR "genetics"[All Fields] OR "genetics"[MeSH Terms]).

**Web of science including MEDLINE and EMBASE**

1. (epilepsy aphasia spectrum and genetics) Timespan: All years. Search language=Auto
